# Time-Qualified Patterns of Variation of PPAR**γ**, DNMT1, and DNMT3B Expression in Pancreatic Cancer Cell Lines

**DOI:** 10.1155/2012/890875

**Published:** 2012-08-26

**Authors:** Valerio Pazienza, Francesca Tavano, Massimo Francavilla, Andrea Fontana, Fabio Pellegrini, Giorgia Benegiamo, Vincenzo Corbo, Fabio Francesco di Mola, Pierluigi Di Sebastiano, Angelo Andriulli, Gianluigi Mazzoccoli

**Affiliations:** ^1^Research Laboratory and Gastroenterology Unit, Scientific Institute and Regional General Hospital “Casa Sollievo della Sofferenza”, 71013 San Giovanni Rotondo, Italy; ^2^Research Laboratory and Surgery Unit, Scientific Institute and Regional General Hospital “Casa Sollievo della Sofferenza”, 71013 San Giovanni Rotondo, Italy; ^3^Computing Unit, Scientific Institute and Regional General Hospital “Casa Sollievo della Sofferenza”, 71013 San Giovanni Rotondo, Italy; ^4^Unit of Biostatistics, Scientific Institute and Regional General Hospital “Casa Sollievo della Sofferenza”, 71013 San Giovanni Rotondo, Italy; ^5^ARC-NET Miriam Cherubini Centre for Applied Research on Cancer, University and Hospital Trust of Verona, 37100 Verona, Italy; ^6^Division of Internal Medicine and Chronobiology Unit, Department of Medical Sciences, Scientific Institute and Regional General Hospital “Casa Sollievo della Sofferenza”, 71013 San Giovanni Rotondo, Italy

## Abstract

Carcinogenesis is related to the loss of homeostatic control of cellular processes regulated by transcriptional circuits and epigenetic mechanisms. Among these, the activities of peroxisome proliferator-activated receptors (PPARs) and DNA methyltransferases (DNMTs) are crucial and intertwined. PPAR**γ** is a key regulator of cell fate, linking nutrient sensing to transcription processes, and its expression oscillates with circadian rhythmicity. Aim of our study was to assess the periodicity of PPAR**γ** and DNMTs in pancreatic cancer (PC). We investigated the time-related patterns of *PPARG, DNMT1*, and *DNMT3B* expression monitoring their mRNA levels by qRT-PCR at different time points over a 28-hour span in BxPC-3, CFPAC-1, PANC-1, and MIAPaCa-2 PC cells after synchronization with serum shock. *PPARG* and *DNMT1* expression in PANC-1 cells and *PPARG* expression in MIAPaCa-2 cells were characterized by a 24 h period oscillation, and a borderline significant rhythm was observed for the *PPARG, DNMT1*, and *DNMT3B* expression profiles in the other cell lines. The time-qualified profiles of gene expression showed different shapes and phase relationships in the PC cell lines examined. In conclusion, *PPARG* and *DNMTs* expression is characterized by different time-qualified patterns in cell lines derived from human PC, and this heterogeneity could influence cell phenotype and human disease behaviour.

## 1. Introduction

Cancer statistics rank pancreatic cancer as the fourth leading cause of malignancy-related death worldwide [1], and incidence and mortality rates are very similar, due to difficult early diagnosis, elevated aggressiveness, and chemotherapy resistance. Bad prognosis and lack of effective treatment are responsible for high lethality, so that there is pressing need to identify molecular biomarkers for prognostic assessment and target therapy. The preservation of tissue integrity is critical for organism survival and relies on tissue renewal, driven by stem cells that are capable of responding to injury and repairing tissue damage, caused by physical, chemicals, microbial, and mutagenic agents. Transcriptional mechanisms regulate cell processes underlying cell renewal and comprising proliferation, differentiation, cell death, and apoptosis. Carcinogenesis relies on the loss of homeostatic mechanisms regulating cell proliferative, differentiative, and survival processes. Among the transcriptional regulators an important role is played by the peroxisome proliferator-activated receptors (PPARs), ligand-activated transcription factors belonging to the superfamily of nuclear hormone receptors, which are considered to be involved in the regulation of nutrient metabolism and energy homeostasis, and in various pathophysiological processes, such as metabolic derangement, inflammation, and cancerogenesis [2]. PPARs are crucial for the transduction of metabolic and nutritional signals into transcriptional responses and comprise three isoforms, PPAR*α*, PPAR*β*/*δ*, and PPAR*γ*, with a high degree of homology but with distinct biological activities [3]. PPAR*α* is mainly involved in lipid metabolism, the function of PPAR*β*/*δ* is not entirely clear, and PPAR*γ* regulates cell fate and differentiation decisions, as well as adipogenesis and fat storage [4–7]. PPAR*γ* expression oscillates over a 24-hour span, and its circadian rhythmicity is crucial in the crosstalk between feeding/fasting cycles, nutrient sensing, metabolic pathways and transcriptional processes. The derangement of this crosstalk is involved in cancer development [8, 9]. High-affinity synthetic ligands, the thiazolidinedione, prompted the study of PPAR*γ* signalling pathways in the regulation of metabolic processes and are currently evaluated as possible therapeutic tools to take advantage of PPAR*γ* prodifferentiative effects in cancer treatment [10]. 

Transcriptional processes are regulated also by epigenetic mechanisms, such as acetylation/deacetylation and methylation/demethylation. DNA methyltransferases (DNMTs) play a critical role in epigenetic mechanisms attaching methyl groups to DNA, and in particular DNMT1 keeps up the methylation pattern during DNA replication, whereas DNMT3a and DNMT3b primarily catalyze *de novo* methylation [11–13]. An intriguing interaction between PPAR*γ* and DNMTs has been recently suggested by the downregulation of DNA methyltransferases evidenced in immune cells following ligand-dependent PPAR*γ* activation [14].

The aim of our study was to assess the time-related patterns of variation of PPAR*γ* and DNMTs in pancreatic cancer using *in vitro* models represented by pancreatic cancer cell lines evaluated after synchronization.

## 2. Material and Methods

### 2.1. Cell Culture and Serum Shock Procedures

BxPC-3, CFPAC-1, PANC-1, and MIA PaCa-2 cells were cultured at 37°C in 5% CO_2_ atmosphere in DMEM medium supplemented with 10% fetal bovine serum (FCS), 100 U/mL penicillin, and 100 ng/mL streptomycin (Invitrogen Life Technologies, Milan, Italy) while CFPAC-1 and MIA PaCa-2 were maintained in RPMI medium (Invitrogen Life Technologies, Milan, Italy). Cell synchronization was obtained by means of serum shock performed as follows: approximately 5 × 10^5^ cells/6 wells were plated the day before the experiments. At the day of the experiments, culture medium was exchanged with serum-rich medium with 50% FBS, and after 2 hours this medium was replaced as described [15]. The cells were harvested over 28 hours at the indicated time points after serum shock.

### 2.2. Quantitative Real-Time Polymerase Chain Reaction

Total RNA was extracted from BxPC-3, CFPAC-1, PANC-1, and MIA PaCa-2 cells at the indicated time points after serum shock using the RNeasy Mini Kit (Qiagen S.P.A. Milano Italy) and subsequently digested by DNase I. cDNA was synthesized from 50 ng total RNA, and quantitative Real-Time PCR (qRT-PCR) was performed using QuantiFast Sybr Green PCR kit following the one-step protocol. For real-time RT-PCR, we used the following SYBR Green QuantiTect Primer purchased from Qiagen: *PPARG* (QT00029841), human *DNMT1* (QT00034335) and *DNMT3B* (QT00032067). Reactions were set up in 96-well plates using a 7700 Real-Time PCR System (Applied Biosystems, Foster City, CA), and all samples were assayed in triplicate. Optical data obtained were analyzed using the default and variable parameters available in the SDS software package (version 1.9.1; Applied Biosystems, Foster City, CA). Expression levels of target gene were normalized using the housekeeping control gene TATA-binding protein (TBP, QT00000721). 

### 2.3. Statistical Analysis

Gene expression values were normalized, for each variable for each cell line, to the expression value of the first time point (T0) of sample collection after serum shock to reduce interassay level variability. Analysis of periodicity patterns was performed, for each time series of the normalized gene expression values, by fitting a least-squares linear regression of a single component (24 h) cosine waveform [16], using the MATLAB statistical package (MathWorks, Natick, Massachusetts, USA). The following parameters were estimated: “mesor” (the overall mean level of the wave); the “amplitude” (*A*, the range from the maximum and the minimum peaks of the best-fitted curve), and the “acrophase” (*a*Ø, the time in angular degrees, from the local midnight Ø, of the wave peak: acro = peak). *P*-values from *F*-statistics were reported for each fitted single cosinor model, to test the null hypothesis of zero amplitude (where the wave has no periodicity). Furthermore, a novel statistical approach was employed to compare the evolution of different time qualified profiles of gene expression in the cell lines, by means of suitable statistical contrasts from a multivariate periodic linear mixed model. In particular, for each comparison, two statistical contrasts were assessed, testing whether the rhythms have an identical or opposing waveform, respectively [17]. The periodic linear mixed model can be thought as the join assessment of many different cosinor models (each one including a specific number of harmonic terms). With respect to cosinor analysis, this novel statistical approach enables the comparison of the evolution of multiple biological rhythms by jointly representing all of them in terms of sine and cosine series into a multivariate linear mixed model, taking into account all their interdependencies (intra- and interoutcome correlation structures), as well as the collection of unequally spaced measures over time and heterogeneity between gene expressions. Moreover, any specific pairwise comparison between the biological rhythms can be performed by means of proper statistical contrasts. *P*-values <0.05 were considered for statistical significance. Statistical analyses were performed using MATLAB and SAS Release 9.1.3 (SAS Institute, Cary, NC).

## 3. Results

**Figure 1:**
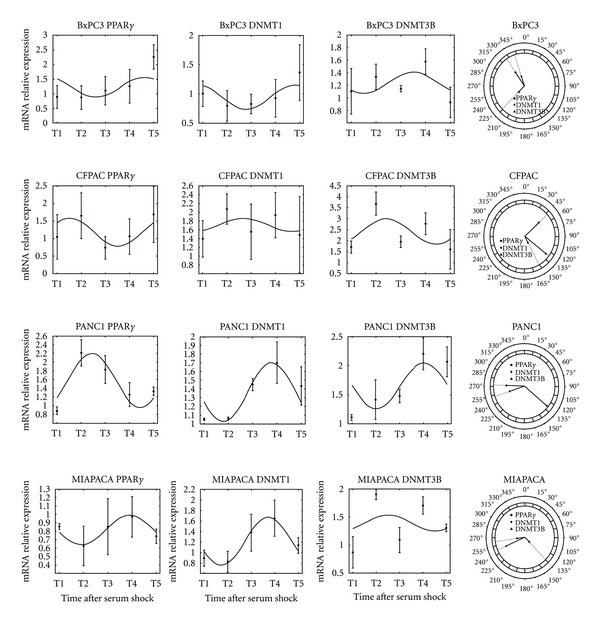
Chronograms displaying time qualified variations of *PPARG*, *DNMT1*, and *DNMT3B* expression level in pancreatic cancer cell lines. Original units standardized to T0 and combined for analyses. Polarograms of cosinor analysis showing the acrophases for the expression values of *PPARG*, *DNMT1*, and *DNMT3B*. Radial axis represents the time point (in degrees) after serum shock corresponding to the acme and vector length represents the amplitude of the oscillation.

Results from cosinor analysis were reported in Table 1 and evidence a clear 24 h periodicity for the time-qualified variations of expression of *PPARG* (*P* = 0.016) and *DNMT1* (*P* = 0.024) in PANC-1 cells and *PPARG* (*P* = 0.010) in MIA PaCa-2 cells, whereas a borderline significant rhythm was observed for the other *PPARG*, *DNMT1,* and *DNMT3B* expression profiles in the examined cell lines (Figure 1). 

**Figure 2:**
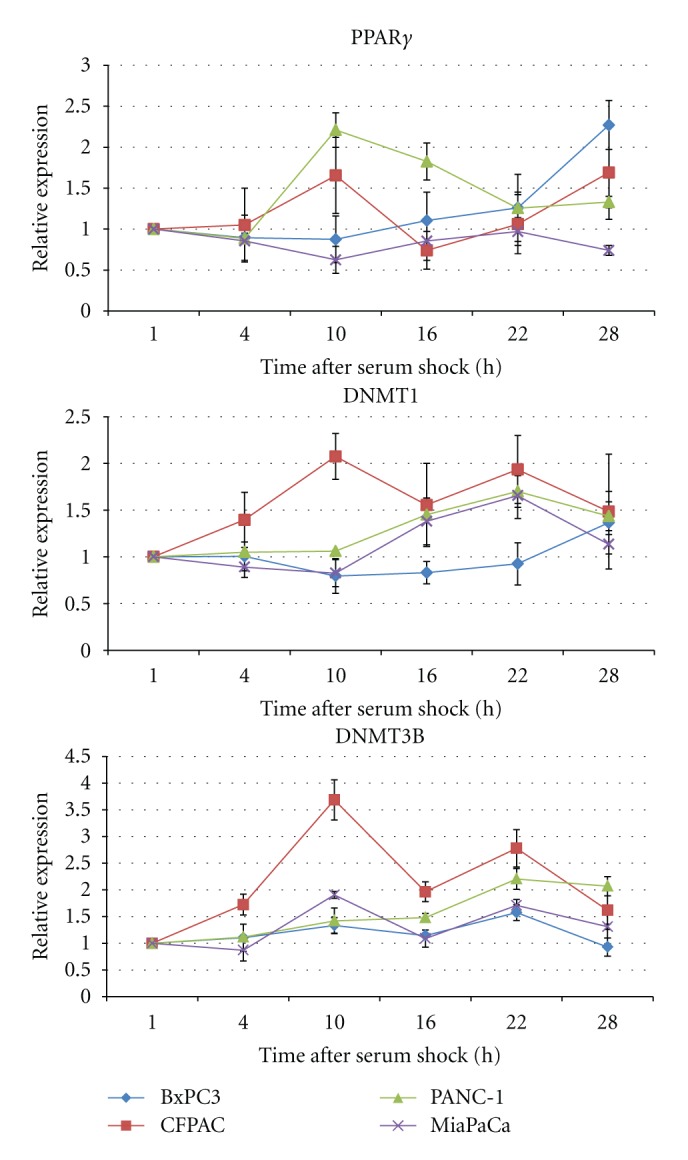
*x*-*y* plots showing the time-related profiles of expression level changes of *PPARG*, *DNMT1*, and *DNMT3B* in pancreatic cancer cell lines. Original units standardized to T0 and combined for analyses. Gene expression data assigned to actual collection time after serum shock.

Results from multivariate periodic regression analysis were reported in Table 2. Pairwise comparisons suggested that in BxPC-3 cells the time profiles of both *PPARG* and *DNMT1* showed flat shapes, whereas the time profiles of *PPARG* and *DNMT3B*, as well as those of *DNMT1* and *DNMT3B*, were opposing. In CFPAC-1 cells the time profiles of all the expressions of *PPARG*, *DNMT1*, and *DNMT3B* were different (neither identical nor opposing). In PANC-1 cells the time profiles of *PPARG* and *DNMT1* were different, the time profiles of *PPARG* and *DNMT3B* were opposing, and the time profiles of *DNMT1* and *DNMT3B* had flat shapes. In MIA PaCa-2 cells the time profiles of all the expressions of *PPARG*, *DNMT1*, and *DNMT3B* were different (neither identical nor opposing) (Figure 2).

## 4. Discussion

Nycthemeral variations with a 24 h periodicity (circadian, from the Latin *circa* and *dies*) characterize behavior and physiology in the greater part of living organisms and contribute to homeostasis maintenance ensuring optimal timing of cellular phenomena in body systems, orchestrated by a complex network of transcriptional circuits [18–20]. Circadian rhythmicity is driven at the body level by a central pacemaker and master oscillator located in the hypothalamic suprachiasmatic nuclei (SCN) entrained by the light/dark cycle *via* the retinohypothalamic tract [21]. At the tissue-specific and single-cell levels the circadian rhythmicity is driven by molecular clocks ticked by transcription/translation feedback loops operated by a set of genes (so-called clock genes: *BMAL1, CLOCK, PER 1–3, CRY 1-2*) and their coded proteins, entrained by the SCN *via* humoral and neural outputs, and in a tissue-specific manner by other factors, such as feeding and temperature fluctuations [22–28]. The biological clocks control cell processes and tissue/organ functions driving the expression of genes coding for transcriptional factors, such as DBP (albumin D-site binding protein) and E4BP4 (adenoviral E4 protein-binding protein), which steer the expression of so-called clock controlled genes and tissue-specific output genes. The transcription factors DBP and E4BP4 among other processes control the circadian rhythmicity of PPAR*γ* by binding to *PPARG *first exon D-sites with functional promoter activity [9].

Disruption of the circadian clock circuitry and alteration of the physiological circadian rhythmicity are considered to be involved in the processes underlying tumorigenesis [29–36].

Considering the important role played in the transcriptional processes by epigenetic mechanisms such as reversible or irreversible attachment of methyl groups to DNA catalyzed by DNMTs [37] and the recently evidenced interaction between PPAR*γ* and DNMTs [14], we sought to evaluate if PPAR*γ* and DNMTs show correspondent oscillation in pancreatic cancer, analyzing their time-related patterns of variation in synchronized pancreatic cancer cell lines. 

Our data put in evidence important differences in the periodicity and in the phase relationships of *PPARG*, *DNMT1*, and *DNMT3B* expression levels among the diverse cell lines examined, maybe related to a different genetic background in the diverse pancreatic cancer cells [38]. 

In the BxPC-3 cell line, mucin-producing cells derived from a human primary pancreatic adenocarcinoma, a borderline significant 24 h periodicity was evidenced for the *PPARG*, *DNMT1*, and *DNMT3B* expression patterns, and the time-qualified profiles of *PPARG* and *DNMT3B*, as well as the time qualified profiles of *DNMT1* and *DNMT3B*, were opposing.

In CFPAC-1 cells, derived from a pancreatic ductal adenocarcinoma liver metastasis of a patient with cystic fibrosis, a borderline significant rhythmicity with a 24 h period was found for the *PPARG*, *DNMT1*, and *DNMT3B* expression patterns, and the time-qualified profiles showed different shapes.

In PANC-1 cells, an epithelial-like cell line derived from a human pancreatic carcinoma, a clear 24 h periodicity was observed for the time qualified variations of *PPARG* and *DNMT1* expression, a borderline significant rhythmicity with a 24 h period was observed for the *DNMT3B* expression pattern, and the time qualified profiles of *PPARG* and *DNMT3B* were opposing, whereas those of *PPARG* and *DNMT1* were different, and the time qualified profiles of *DNMT1* and *DNMT3B* showed flat shapes.

In the MIA PaCa-2 cell line, established from a human pancreatic adenocarcinoma, a clear 24-h periodicity was observed for the time qualified variations of expression of *PPARG*, and a borderline significant rhythmicity with a 24 h period was observed for the *DNMT1* and *DNMT3B* expression patterns, and the time qualified profiles of *PPARG* and *DNMT1* as well as those of *PPARG* and *DNMT3B* and the time qualified profiles of *DNMT1* and *DNMT3B* were different (neither identical nor opposing).

The different time qualified profiles and phase relationships evidenced in the pancreatic cancer cell lines examined suggest that they rely on a dissimilar temporal architecture of transcriptional circuits and epigenetic mechanisms, which may influence cancer cell behavioral phenotype and possibly response to therapy.

Normal pancreatic duct epithelial cells do not seem to express PPAR*γ*, whereas human pancreatic cancer cell lines express the nuclear receptor, and drugs of the thiazolidinedione class transactivate the transcription of a peroxisome proliferator response element-driven promoter in a dose-dependent fashion [39]. Besides, immunohistochemical staining of resected specimens by means of a polyclonal PPAR*γ* antibody has evidenced PPAR*γ* protein expression in the nuclei of carcinoma cells in 90% of human pancreatic adenocarcinomas [40]. Selective PPAR*γ* ligands inhibit pancreatic cancer cell growth in a dose-dependent manner and reduce the invasiveness of the tumor cells, suggesting a potential role for these agents in the adjuvant treatment of pancreatic cancer [41]. Furthermore, the first-line drug for the treatment of unresectable pancreatic cancer is represented by the nucleoside analog gemcitabine, and PPAR*γ* ligands potentiate its cytotoxic action on human pancreatic cancer cells in a dosage-dependent manner and are tested to improve the prognosis of pancreatic cancer patients [42].

Inactivation of tumor suppressor genes is central to the development of all common forms of human cancer, and this inactivation often results from epigenetic silencing rather than intragenic mutations. A prevalent mechanism of tumor-suppressor gene inactivation in neoplastic disease is represented by transcriptional silencing by CpG island methylation, and the prototypic DNA methyltransferase, DNMT1, accounts for most methylation in mouse cells, but human cancer cells lacking DNMT1 retain significant genomic methylation and associated gene silencing [11]. In human cells, the mechanisms underlying locus-specific or global methylation patterns remain unclear, but genetic disruption of both DNMT1 and DNMT3b nearly eliminates methyltransferase activity and reduces genomic DNA methylation by greater than 95%. The importance of the DNA methyltransferase DNMT1 for the maintenance of cell methylation and its role in tumorigenesis have been highlighted by genetic experiments. DNMT1 is necessary and sufficient to maintain global methylation and aberrant CpG island methylation in human cancer cells, and selective depletion of DNMT1 with antisense inhibitors has been shown to induce demethylation and reactivation of silenced tumor-suppressor genes such as *CDKN2A*. Inactivation of both *DNMT1* and *DNMT3B* induces low levels of DNA methylation, whereas selective deletion of *DNMT1* alleles in cancer cells produces clones that retain CpG island methylation and associated tumor-suppressor gene silencing, suggesting that the two DNMTs cooperatively maintain DNA methylation and gene silencing in human cancer cells, providing convincing support that such methylation is indispensable for best possible neoplastic proliferation [11, 35].

In conclusion, the cell lines derived from human pancreatic cancers are characterized by different arrays of time qualified profiles of gene expression and epigenetic modifications, which could be related to particular genetic backgrounds and could impinge on cancer cell phenotype, suggesting variable temporal organization of cell processes that could conditionate disease behaviour and response to timed delivery of conventional chemotherapy.

## Figures and Tables

**Table 1:** Rhythm parameters from fitted single cosinor models for mRNA expression calculated on original values normalized to the first time point of sample collection after serum shock.

BxPC3			
	*PPARG*	*DNMT1*	*DNMT3B*
Mesor	1.22	0.94	1.24
Amplitude	0.33	0.20	0.16
Acrophase	325.32	342.87	225.6
*P*-value	0.780	0.430	0.722

CFPAC			
	*PPARG*	*DNMT1*	*DNMT3B*

Mesor	1.18	1.71	2.42
Amplitude	0.40	0.14	0.57
Acrophase	47.58	151.69	127.57
*P*-value	0.446	0.839	0.753

PANC1			
	*PPARG*	*DNMT1*	*DNMT3B*

Mesor	1.58	1.36	1.65
Amplitude	0.62	0.33	0.39
Acrophase	129.72	251.25	272.44
*P*-value	0.016	0.024	0.630

MIAPACA			
	*PPARG*	*DNMT1*	*DNMT3B*

Mesor	0.81	1.21	1.39
Amplitude	0.17	0.45	0.14
Acrophase	261.86	245.4	136.43
*P*-value	0.010	0.067	0.933

Overall gene expression levels were analyzed for time effect across the timepoints by single cosinor: fit of 24 h cosine to all data by least squares linear regression. Acrophase, the crest time of rhythm, is expressed in degrees. *P*-values refer to test for time effect (zero amplitude).

**Table:** (a)

		BxPC3 statistical contrasts
Hypotheses	*F*-value	*P*-value
H_01_: Biorhythms of *PPARG* and *DNMT1* are identical	0.57	0.695
H_02_: Biorhythms of *PPARG* and *DNMT1* are opposing	2.11	0.189
H_01_: Biorhythms of *PPARG* and *DNMT3B* are identical	4.16	0.039
H_02_: Biorhythms of *PPARG* and *DNMT3B* are opposing	0.46	0.764
H_01_: Biorhythms of *DNMT1* and *DNMT3B* are identical	11.67	0.001
H_02_: Biorhythms of *DNMT1* and *DNMT3B* are opposing	1.61	0.246

		Decisions derived from statistical contrasts

		(1) Biorhythms of *PPARG* and *DNMT1* have flat shapes, although statistical tests slightly suggest that they could be identical (i.e., no sufficient statistical power). (2) Biorhythms of *PPARG* and *DNMT3B* are opposing. (3) Biorhythms of *DNMT1 * and *DNMT3B* are opposing

		CFPAC statistical contrasts
Hypotheses	*F*-value	*P*-value

H_01_: Biorhythms of *PPARG* and *DNMT1* are identical	7.82	0.003
H_02_: Biorhythms of *PPARG* and *DNMT1* are opposing	6.70	0.021
H_01_: Biorhythms of *PPARG* and *DNMT3B* are identical	43.78	<0.001
H_02_: Biorhythms of *PPARG* and *DNMT3B* are opposing	22.68	<0.001
H_01_: Biorhythms of *DNMT1* and *DNMT3B* are identical	28.98	<0.001
H_02_: Biorhythms of *DNMT1* and *DNMT3B* are opposing	38.02	<0.001

		Decisions derived from statistical contrasts

		(1) Biorhythms of *PPARG* and *DNMT1* are different (neither identical nor opposing). (2) Biorhythms of *PPARG* and *DNMT3B* are different (neither identical nor opposing). (3) Biorhythms of *DNMT1* and *DNMT3B* are different (neither identical nor opposing)

		PANC1 statistical contrasts
Hypotheses	*F*-value	*P*-value

H_01_: Biorhythms of *PPARG* and *DNMT1* are identical	52.22	<0.001
H_02_: Biorhythms of *PPARG* and *DNMT1* are opposing	5.78	0.010
H_01_: Biorhythms of *PPARG* and *DNMT3B* are identical	16.14	0.002
H_02_: Biorhythms of *PPARG* and *DNMT3B* are opposing	3.16	0.070
H_01_: Biorhythms of *DNMT1* and *DNMT3B* are identical	1.96	0.196
H_02_: Biorhythms of *DNMT1* and *DNMT3B* are opposing	3.57	0.063

		Decisions derived from statistical contrasts

		(1) Biorhythms of *PPARG* and *DNMT1* are different (neither identical nor opposing). (2) Biorhythms of *PPARG* and *DNMT3B * are opposing, although statistical tests slightly suggest that they could be different at all. (3) Biorhythms of *DNMT1* and *DNMT3B* have flat shapes, although statistical tests slightly suggest that they could be identical

		MIAPACA statistical contrasts
Hypotheses	*F*-value	*P*-value

H_01_: Biorhythms of *PPARG* and *DNMT1* are identical	11.63	<0.001
H_02_: Biorhythms of *PPARG* and *DNMT1* are opposing	7.23	0.018
H_01_: Biorhythms of *PPARG* and *DNMT3B* are identical	9.89	0.001
H_02_: Biorhythms of *PPARG* and *DNMT3B* are opposing	8.52	0.003
H_01_: Biorhythms of *DNMT1* and *DNMT3B* are identical	17.71	<0.001
H_02_: Biorhythms of *DNMT1* and *DNMT3B* are opposing	9.06	0.002

		Decisions derived from statistical contrasts

		(1) Biorhythms of *PPARG* and *DNMT1* are different (neither identical nor opposing). (2) Biorhythms of *PPARG* and *DNMT3B* are different (neither identical nor opposing). (3) Biorhythms of *DNMT1* and *DNMT3B* are different (neither identical nor opposing)


**Table:** (b)

			H_01_ *“identical biorhythms” *
		Rejected	Not-rejected
	Rejected	The biorhythms are different (neither identical nor opposing)	The biorhythms are identical
H_02 _ *“opposing biorhythms” *	Not-rejected	The biorhythms are opposing	The biorhythms have flat shape
